# Predictors and outcomes of shunt-dependent hydrocephalus in patients with aneurysmal sub-arachnoid hemorrhage

**DOI:** 10.1186/1471-2482-12-12

**Published:** 2012-07-05

**Authors:** Yi-Min Wang, Yu-Jun Lin, Ming-Jung Chuang, Tsung-Han Lee, Nai-Wen Tsai, Ben-Chung Cheng, Wei-Che Lin, Ben Yu-Jih Su, Tzu-Ming Yang, Wen-Neng Chang, Chih-Cheng Huang, Chia-Te Kung, Lian-Hui Lee, Hung-Chen Wang, Cheng-Hsien Lu

**Affiliations:** 1 Division of Neurosurgery, Department of Surgery, Yuan's General Hospital, Kaohsiung, Taiwan; 2 Department of Neurosurgery, Kaohsiung Chang Gung Memorial Hospital, Chang Gung University College of Medicine, Kaohsiung, Taiwan; 3 Department of Biological Science, National Sun Yat-Sen University, Kaohsiung, Taiwan; 4 Department of Neurology, Kaohsiung Chang Gung Memorial Hospital, Chang Gung University College of Medicine, 123, Ta Pei Road, Niao Sung district, Kaohsiung 83304, Taiwan; 5 Department of Medicine, Kaohsiung Chang Gung Memorial Hospital, Chang Gung University College of Medicine, Kaohsiung, Taiwan; 6 Department of Radiology, Kaohsiung Chang Gung Memorial Hospital, Chang Gung University College of Medicine, Kaohsiung, Taiwan; 7 Department of Emergency Medicine, Kaohsiung Chang Gung Memorial Hospital, Chang Gung University College of Medicine, Kaohsiung, Taiwan

**Keywords:** Outcome, Risk factors, Hydrocephalus after spontaneous aneurysmal subarachnoid hemorrhage

## Abstract

**Background:**

Hydrocephalus following spontaneous aneurysmal sub-arachnoid hemorrhage (SAH) is often associated with unfavorable outcome. This study aimed to determine the potential risk factors and outcomes of shunt-dependent hydrocephalus in aneurysmal SAH patients but without hydrocephalus upon arrival at the hospital.

**Methods:**

One hundred and sixty-eight aneurysmal SAH patients were evaluated. Using functional scores, those without hydrocephalus upon arrival at the hospital were compared to those already with hydrocephalus on admission, those who developed it during hospitalization, and those who did not develop it throughout their hospital stay. The Glasgow Coma Score, modified Fisher SAH grade, and World Federation of Neurosurgical Societies grade were determined at the emergency room. Therapeutic outcomes immediately after discharge and 18 months after were assessed using the Glasgow Outcome Score.

**Results:**

Hydrocephalus accounted for 61.9% (104/168) of all episodes, including 82 with initial hydrocephalus on admission and 22 with subsequent hydrocephalus. Both the presence of intra-ventricular hemorrhage on admission and post-operative intra-cerebral hemorrhage were independently associated with shunt-dependent hydrocephalus in patients without hydrocephalus on admission. After a minimum 1.5 years of follow-up, the mean Glasgow outcome score was 3.33 ± 1.40 for patients with shunt-dependent hydrocephalus and 4.21 ± 1.19 for those without.

**Conclusions:**

The presence of intra-ventricular hemorrhage, lower mean Glasgow Coma Scale score, and higher mean scores of the modified Fisher SAH and World Federation of Neurosurgical grading on admission imply risk of shunt-dependent hydrocephalus in patients without initial hydrocephalus. These patients have worse short- and long-term outcomes and longer hospitalization.

## Background

Aneurysmal sub-arachnoid hemorrhage (SAH) still has high mortality and morbidity rates despite modern neurosurgical techniques, new powerful imaging modalities, and care of such patients [1]. An important neurologic complication is hydrocephalus [2-5], which can be either acute-onset on admission or progressive during the hospital stay [2-5]. The overall risk of hydrocephalus after aneurysmal SAH varies between 6% to 67% in different series [6,7] although only 10-20% of them will require permanent CSF diversion [6,7]. To date, no clinical study has focused specifically on predicting shunt dependency in patients with aneurysmal SAH but without hydrocephalus upon arriving at the hospital, or the outcome of these specific patients for a longer follow-up period. Because of possible benefits of therapeutic intervention, there is a need for better delineation of the potential risk factors and clinical features in this specific sub-group.

This study aimed to analyze the clinical features, neuro-imaging findings, and clinical scores and measurements to determine the potential risk factors predictive of shunt-dependent hydrocephalus in patients with aneurysmal SAH but without hydrocephalus upon arriving at the hospital. The study also compared these patients to those with hydrocephalus at the time of admission, those who developed it during hospitalization, and those who did not develop it after 1.5 years of follow-up.

## Methods

### Study design

From January 2003 to December 2005, 168 SAH patients admitted to the Department of Neurosurgery at the Chang Gung Memorial Hospital in Kaohsiung were enrolled. Chang Gung Memorial Hospital-Kaohsiung is a 2482-bed acute-care teaching hospital, which is the largest medical center in the southern part of Taiwan providing both primary and tertiary referral care to patients. All patients received complete medical and neurologic examinations, and brain computed tomography (CT) with cerebral angiography. The Chang Gung Memorial Hospital hospital’s Institutional Review Committee on Human Research approved the study (Institutional Review Board numbers: 96-1575B). Neurosurgeons and neuro-radiologists integrated the clinical manifestations and neuro-imaging findings.

### Diagnostic criteria of spontaneous aneurysmal sub-arachnoid hemorrhage

All of the patients received brain CT scans soon after arrival at the emergency room, and follow-up brain CT post-surgery. Emergency brain CT scans were done if there was clinical deterioration, including acute-onset focal neurologic deficits, seizures or status epilepticus, or progressively disturbed consciousness and post-neurosurgical procedures.

In the study hospital, it was routine practice to arrange cerebral angiograms immediately after hospitalization. A ruptured, angiographically verified aneurysm was the cause of the SAH in all patients. Patients initially treated in other hospitals but subsequently transferred for further therapy were also included in the study and their initial clinical and laboratory data at the previous hospital were used for analysis. Patients were excluded if: 1) the initial angiogram was negative for SAH; 2) they suffered from non-aneurysmal SAH, such as traumatic SAH; 3) they were comatose or were considered unlikely to survive for more than one week; and 4) there were pre-existing neurologic deficits.

### Clinical assessment

Hydrocephalus was judged retrospectively by a dilated temporal horn of the ventricle without obvious brain atrophy and/or an Evan’s ratio >0.3 on initial CT scan. The Evan’s ratio was the ratio of the ventricular width of the bilateral frontal horn to the maximum bi-parietal diameter [8]. Furthermore, shunt-dependent hydrocephalus was defined as clinical symptoms of hydrocephalus (i.e., decreased mental status, axial rigidity, and incontinence) with radiographic evidence of enlarged ventricles or high opening pressure on repeated lumbar punctures requiring the insertion of a ventriculo-peritoneal (VP) shunt [2,3].

The characteristics and circumstances, and complications following underlying SAH or treatment were documented. The diagnosis of acute symptomatic cerebral infarction following aneurysmal SAH was based on both new-onset cerebral infarctions (on follow-up brain CT) and the presence of acute neurologic deficits causally related to the cerebral infarction. Patients were considered to have multiple infarctions if at least two locations with infarctions were found. Re-bleeding was defined as sudden deterioration of the clinical state accompanied by new or increased blood on brain CT scan [9]. Symptomatic vasospasm was defined as both the development of focal neurologic signs or deterioration in conscious state and evidence of vasospasm or presence of stenotic flow velocity shown by trans-cranial color-coded sonography through cerebral angiogram, CT angiography, or magnetic resonance angiography [10,11]. All diagnoses of hydrocephalus, re-bleeding, and vasospasm were based on brain CT evidence.

The Glasgow Coma Score (GCS) [12], modified Fisher SAH grade [13], and World Federation of Neurosurgical Societies (WFNS) grade [14] were determined by neurosurgeons upon the patient’s arrival at the emergency room. Evaluation of therapeutic outcome both immediately after discharge and 18 months after used Glasgow Outcome Score (GOS). The follow-up period was terminated by death or by the end of the study (June 2007). The outpatient department followed-up most patients after discharge as part of standard care, while others were interviewed by telephone to identify neurologic outcome.

### Statistical analysis

Three separate series of statistical analyses were performed. First, to compare demographic data among patients who already had hydrocephalus at the time of admission, those who developed it during hospitalization and those who did not have it during the hospital stay, categorical variables were assessed by Chi-square test, and continuous variables were logarithmically transformed to improve normality and compared using one-way ANOVA for parametric data, followed by Scheffe’s multiple comparison for post-hoc test for significant pairwise differences.

Second, risk factors of shunt-dependent hydrocephalus in patients with aneurysmal SAH but without hydrocephalus upon arrival were analyzed. Baseline clinical data, including gender, clinical manifestations, and neuro-imaging findings between those with and those without shunt-dependent hydrocephalus were analyzed by Chi-square test or Fisher’s exact test, where appropriate. The mean ages, mean systolic and diastolic pressure, and mean hospitalization days between the two patient groups were analyzed by Student’s *t*-test. The GCS at the time of admission, GOS at the time of discharge and 18 months after discharge, mean modified Fisher SAH grade, and mean WFNS grade between the two patient groups were analyzed by the Wilcoxon rank sum test.

Lastly, stepwise logistic regression was used to evaluate the relationships between clinical factors and the presence of shunt-dependent hydrocephalus, with adjustments for other potential confounding factors. All of the statistical analyses was conducted using the SAS software package, version 9.1 (2002, SAS Statistical Institute, Cary, North Carolina).

## Results

### Baseline characteristics of the study patients

Of the 168 aneurysmal SAH patients (52 males and 116 females), 104 had complications with hydrocephalus during the acute phase, including initial hydrocephalus in 82 and subsequent hydrocephalus in 22. Their characteristics in terms of hydrocephalus and location and seize of aneurysms were listed in Table 1 and 2. Hypertension, diabetes mellitus (DM), and coronary artery diseases were the three most common underlying diseases. The proportions of nosocomial pneumonia in patients with initial hydrocephalus and subsequent hydrocephalus were 39% (32/82) and 50% (11/22), respectively.

**Table 1:** Characteristics of patients with aneurysmal SAH in terms of hydrocephalus (n = 168)

		**With Hydrocephalus**		**Without Hydrocephalus**	**P value**
		**Initial hydrocephalus**	**Subsequent hydrocephalus**		
		**N = 82**	**N = 22**	**N = 64**	
Mean age, years		57.79 ± 14.79	57.32 ± 11.59	52.59 ± 12.08	0.06
Sex (male/female)		29/53	7/15	16/48	0.403
Mean blood pressure on presentation					
Systolic Blood pressure (mmHg)		148.07 ± 23.62	143.68 ± 24.29	145.73 ± 22.90	0.687
	Diastolic Blood pressure (mmHg)	81.27 ± 13.93	79.13 ± 16.24	81.89 ± 11.87	0.712
	Mean GCS on presentation	10.88 ± 4.07	11.64 ± 3.65	13.16 ± 2.96	0.001^α^
Mean modified Fisher SAH grade on presentation		3.17 ± 0.86	2.82 ± 0.96	2.42 ± 0.79	<0.0001^β^
Mean WFNS grade on presentation		2.95 ± 1.41	2.86 ± 1.46	2.01 ± 1.20	<0.0001^γ^
Mean Hospitalization days		30.40 ± 21.97	44.45 ± 24.34	20.03 ± 16.80	<0.0001^ϵ^
Underlying diseases					
	Atrial fibrillation	2	0	1	
	Coronary artery diseases	3	3	3	0.174
	Diabetes mellitus	8	3	4	06540
	End-stage renal diseases	3	0	2	0.666
	Hypertension	38	12	21	0.119
Treatment^θ^					
	Clipping of aneurysm only	35	17	35	
	Transarterial embolization only	29	3	24	
	Both transarterial embolization and clipping	8	1	1	
	External ventral drainage	52	16	--	
	Ventriculoperitoneal shunt^ι^	32	15	--	
Mean GOS at discharge		3.18 ± 1.34	2.86 ± 0.91	3.97 ± 1.14	<0.0001^η^
	Good recovery	12	0	24	
	Moderate disability	32	6	26	
	Severe disability	11	7	6	
	Vegetative state	13	7	4	
	Death	14	2	4	
Mean GOS after more than 18 months of follow-up		3.70 ± 1.69	3.18 ± 1.26	4.36 ± 1.13	0.002^θ^

Abbreviations: SAH, sub-arachnoid hemorrhage; GCS, Glasgow Coma Scale; GOS, Glasgow Outcome Scale; WFNS, World Federation of Neurosurgical Societies; θ, Not all patients have every treatment; --, not done; ι, Shunt-dependent hydrocephalus; IH, Initial hydrocephalus; SH, Subsequent hydrocephalus; WH, Without Hydrocephalus.

Post-hoc test: α = IH vs. WH, p = 0.001; β = IH vs. WH, p < 0.0001; γ = IH vs. WH, p < 0.0001; SH vs. WH, p = 0.041; ϵ = IH vs. SH, p = 0.019; IH vs. WH, p = 0.011; SH vs. WH, p < 0.001; η = IH vs. WH, p = 0.001; SH vs. WH, p = 0.002; θ = IH vs. WH, p = 0.025; SH vs. WH, p = 0.005.

**Table 2:** Location and seize of aneurysms in patients in terms of hydrocephalus (n = 168)

		**With Hydrocephalus**		**Without Hydrocephalus**	**Total**
		**Initial hydrocephalus**	**Subsequent hydrocephalus**		
		**N = 82**	**N = 22**	**N = 64**	**N = 168**
Location of aneurysm					
Single (n = 156)					
	Anterior communicating artery aneurysm	19	8	24	51
	Posterior communicating artery aneurysm	21	3	7	31
	Middle cerebral artery aneurysm	8	3	10	21
	Internal carotid artery aneurysm	8	3	8	19
	Vertebral artery	4	1	3	8
	Others^η^	16	3	7	26
	Multiple sites (N = 12)	6	1	5	12
	Diameter of aneurysm (mm)^Ф^	0.75 ± 0.20	0.71 ± 0.25	0.67 ± 0.32	0.68 ± 0.31
Shape of aneurysm^Є^					
	Pouch	54	17	52	133
	Lobulation	13	3	8	24
	Fusiform	9	2	1	12
	dissection	4	0	2	6
	Wide neck	2	0	1	3

η = The other locations of aneurysms included the superior cerebellar artery aneurysm in four,, posterior inferior cerebellar artery aneurysm in four, anterior cerebral artery aneurysm in four, pericallosal artery aneurysm in three, posterior cerebral artery aneurysm in four, ophthalmic artery in one, basilar artery aneurysm in five, and anterior inferior cerebellar artery aneurysm in one.

Ф = Indicates the maximum diameter of the aneurysm if at least two aneurysms are found.

Є = Indicates the largest aneurysm if at least two aneurysms are found.

The mean GCS on presentation were 10.88 ± 4.07, 11.64 ± 3.65, and 13.16 ± 2.96 for patients with initial hydrocephalus, subsequent hydrocephalus, and no hydrocephalus, respectively (*p* = 0.001). The mean modified Fisher SAH grade on presentation were 3.17 ± 0.86, 2.82 ± 0.96, and 2.42 ± 0.79, respectively (*p* < 0.0001), while the mean WFNS grade on presentation were 2.95 ± 1.41, 2.86 ± 1.46, and 2.01 ± 1.20, respectively (*p* < 0.0001). The median time (interquartile range) of ventriculostomy insertion relative to the date of presentation were 1 (0, 2) and 1.5 (0.25-6.25) days for patients with initial hydrocephalus and subsequent hydrocephalus, respectively (p = 0.138, Mann–Whitney *U* test).

### Complications following aneurysmal SAH

Complications following underlying aneurysmal SAH among the three patient groups were listed in Table 3. The proportions of intra-ventricular hemorrhage were 51.2% (42/82), 27.2% (6/22), and 7.8% (5/64) in patients with initial hydrocephalus, subsequent hydrocephalus, and no hydrocephalus, respectively (*p* < 0.0001). The proportions of hyponatremia were 12.2% (10/82), 22.7% (5/22), and 3.1% (2/64), respectively (*p* = 0.022), while the proportions of diabetes inspidus were 1.2% (1/82), 9% (2/22), and 0% (0/64), respectively (*p* = 0.018). Other complications following the aneurysmal SAH included cerebral infarctions, aneurysmal re-bleeding, vasospasm, intra-cerebral hemorrhage, and arrhythmia (Table 2).

**Table 3:** Complications following treatment or underlying SAH

		**With Hydrocephalus**		**Without Hydrocephalus**	**P-value**
		**Initial hydrocephalus**	**Subsequent hydrocephalus**		
		**N = 82**	**N = 22**	**N = 64**	
Complications following underlying SAH					
	Cerebral infarctions	18	7	13	0.528
	Vasospasm	16	4	8	0.518
	Rebleeding during hospitalization	10	3	3	0.241
	Seizure	11	5	9	0.537
	Diabetes inspidus	1	2	0	0.018
	Hyponatremia	10	5	2	0.022
	Arrhythmia	1	1	1	0.570
	Intracerebral hemorrhage	14	4	11	0.992
	Intraventricular hemorrhage	42	6	5	<0.0001
	Pneumonia	21	9	4	0.001
	Postoperative intracerebral hemorrhage	5	6	4	0.005
	Shunt infections	5	2	--	
	Over-shunting	1	2	--	
	Shunt obstruction	12	6	--	

Abbreviations: SAH, sub-arachnoid hemorrhage;--, not done.

Complications following the treatment of aneurysmal SAH were listed in Table 2. The proportions of nosocomial pneumonia were 25.6% (21/82), 40.9% (9/22), and 6.3% (4/64) in patients with initial hydrocephalus, subsequent hydrocephalus, and no hydrocephalus, respectively (*p* = 0.001), while the proportions of post-operative intra-cerebral hemorrhage following surgical interventions were 6.1% (5/82), 27.3% (6/22), and 6.3% (4/64), respectively (*p* = 0.005). Complications related to ventriculo-peritoneal (VP) shunt procedures included shunt infections, over-shunting and shunt obstructions (Table 2).

The mean lengths of hospitalization among the three groups were 30.40 ± 21.97, 44.45 ± 24.34, and 20.03 ± 16.80 (*p* < 0.0001). Therapeutic outcomes among the 168 patients after discharge as determined by GOS were 36 normal (21.4%, 36/168), 64 moderate disability (38.1%, 64/168), 24 severe disabilities (14.2%, 24/168), 24 persistent vegetative states (14.2%, 24/168), and 20 mortalities (11.9%, 20/168). The mean GOS score among the three groups were 3.18 ± 1.34, 2.86 ± 0.91, and 3.97 ± 1.14 in patients with initial hydrocephalus, subsequent hydrocephalus, and no hydrocephalus, respectively (*p* < 0.0001). After a 1.5-year follow-up, the mean GOS score among the three groups were 3.70 ± 1.69, 3.18 ± 1.26 and 4.36 ± 1.13, respectively (*p* = 0.002).

### Risk factors of shunt-dependent hydrocephalus

Risk factors of shunt-dependent hydrocephalus in patients with aneurysmal SAH but without hydrocephalus upon arrival at the hospital were listed in Table 4. Statistical analysis revealed significant mean GCS on presentation (*p* = 0.01), mean modified Fisher SAH grade on presentation (*p* = 0.039), mean WFNS grade on presentation (*p* = 0.012), presence of intra-ventricular hemorrhage on admission (*p* < 0.003), and post-operative intra-cerebral hemorrhage (*p* = 0.013). These variables were then used in the stepwise logistic regression model. After analysis, only the presence of intra-ventricular hemorrhage on admission (*p = 0.003*, OR = 9.608, 95% CI: 2.207-41.822) and post-operative intra-cerebral hemorrhage (*p = 0.011*, OR = 7.354, 95% CI: 1.576-34.313) were independently associated with the presence of shunt-dependent hydrocephalus.

**Table 4:** Risk factors of shunt-dependent hydrocephalus in aneurysmal SAH patients without hydrocephalus upon arrival at the hospital

		**Without shunt-dependent hydrocephalus N = 71**	**With shunt-dependent hydrocephalus N = 15**	**P value**	**OR**	**95% CI**
Sex (male/female)		20/51	3/12	0.384	0.638	0.163-2.501
Mean age at onset		52.92 ± 11.85	57.93 ± 12.66	0.146		
Mean blood pressure on presentation						
	Mean Systolic Blood pressure (mmHg)	144.59 ± 22.92	148.13 ± 24.77	0.593		
	Mean diastolic Blood pressure (mmHg)	81.84 ± 12.734	78.07 ± 14.67	0.312		
Mean GCS on presentation		13.17 ± 2.82	10.87 ± 4.17	0.01		
Mean modified Fisher SAH grade on presentation		2.44 ± 0.80	2.93 ± 0.96	0.039		
Mean WFNS grade on presentation		2.07 ± 1.20	3.00 ± 1.60	0.012		
Mean Hospitalization days		24.13 ± 21.50	36.46 ± 20.24	0.0.045		
Neuroimaging findings						
	Rebleeding of aneurysm	4	2	0.280	2.577	0.427-15.563
	Intraventricular hemorrhage on admission	5	6	0.003	8.8	2.223-34.842
	Intracerebral hemorrhage on admission	12	3	0.72	1.229	0.3-5.301
Underlying diseases						
	Hypertension	27	6	1.0	1.086	0.348-3.393
	Atrial fibrillation	1	0	1.0	0.824	0.746-0.909
	Coronary artery diseases	5	1	1.0	0.943	0.102-8.708
	Diabetes mellitus	4	3	0.098	4.188	0.83-21.12
	End stage renal diseases	2	0	1.0	0.821	0.743-0.908
Other complications following aneurysmal SAH						
	Cerebral infarction					
	Symptomatic vasospasm	10	2	1.0	0.938	0.184-4.799
	Seizure	9	5	0.063	3.444	0.957-12.40
	Diabetes inspidus	0	2	0.029	0.155	0.094-0.255
	Hyponatremia	5	2	0.60	2.031	0.355-11.62
	Shunt infection	1	1	0.32	5.0	0.295-84.776
	Postoperative intracerebral hemorrhage	5	5	0.013	6.6	1.617-26.945
	Arrhythmia	1	1	0.320	5.0	0.295-84.776
Outcome						
	Mean Hospitalization days	24.13 ± 21.50	36.47 ± 20.24	0.0452		
	Mean GOS at discharge	3.86 ± 1.16	2.93 ± 1.03	0.014		
	Mean GOS after more than 18 months of follow-up	4.21 ± 1.19	3.33 ± 1.40	0.211		

Abbreviations: N, number of cases; OR, odds ratio; CI, confidence interval; SAH, sub-arachnoid hemorrhage; GCS, Glasgow Outcome Scale; GOS, Glasgow Outcome Scale; WFNS, World Federation of Neurosurgical Societies.

## Discussion

To date, this is the first study to determine the potential risk factors that are predictive of shunt-dependent hydrocephalus in patients with aneurysmal SAH but without hydrocephalus upon arriving at the hospital. Differences in the relative prevalence of hydrocephalus following aneurysmal SAH vary with case ascertainment and inclusion criteria, timing and methods of neuro-imaging studies, serial follow-up neuro-imaging studies, surgical procedure, and presence of complications [1-7]. In the current study, hydrocephalus accounts for 61.9% (104/168) of all episodes, including 82 with initial hydrocephalus on admission and 22 with subsequent hydrocephalus. Such figures are higher than those of two recent studies [3,6] and the largest study [5].

The present study examined the risk factors and outcome of shunt-dependent hydrocephalus in aneurysmal SAH patients and produced two major findings. First, the presence of intra-ventricular hemorrhage, lower mean score of Glasgow Coma Scale, higher mean scores of both the modified Fisher SAH grade and the World Federation of Neurosurgical grade on admission, and complications with post-operative intra-cerebral hemorrhage are significant risk factors for shunt-dependent hydrocephalus in patients without hydrocephalus on admission. Second, shunt-dependent hydrocephalus patients have worse short- and long-term outcomes and longer duration of hospitalization.

For research on the risk factors and outcomes of shunt-dependent hydrocephalus, most large studies have focused on acute or chronic hydrocephalus together, [2,3,6]. Very few have examined both clinical features and outcomes for acute and subsequent hydrocephalus, respectively [4]. The pathogenesis of acute hydrocephalus is thought to result from blockage of CSF flow, producing a pressure gradient, and ultimately leading to enlarged ventricles, whereas the pathogenesis of chronic hydrocephalus involves arachnoid adhesions formed as a result of meningeal reaction to blood products, impairing CSF absorption at the basal cisterns [15,16].

The presence of hydrocephalus does not always lead to the development of shunt dependency although it is a strong predictor of such, as noted in previous studies [17,18] and in the current study. The data here demonstrates that 39% of patients with acute hydrocephalus on admission and 50% of those with subsequent hydrocephalus have undergone permanent shunting procedures. Furthermore, there is evidence in literature suggesting that aggressive external ventricular drainage significantly reduces the need for permanent shunting among these patients [19]. Although the effect of temporary ventriculostomy placement on the development of hydrocephalus is not studied, its effects on the outcome of hydrocephalus may also be considered in future studies.

Several studies demonstrate a strong relationship between poor levels of consciousness on admission and hydrocephalus [5,7]. Both acute and subsequent hydrocephalus cases also have similar results. Some studies show that the amount of blood in the sub-arachnoid space has special significance [5,7] while the current study demonstrates higher mean modified Fisher SAH grade on presentation in patients who have shunt-dependent hydrocephalus. The effect of intra-ventricular hemorrhage on the development of hydrocephalus is also well established [5,7]. Some authors suggest that the presence of blood clots and high CSF viscosity can lead to an obstructive form of hydrocephalus and early CSF circulation disturbances [20,21]. In the current series, intra-ventricular hemorrhage is a significant risk factor for the development of shunt-dependent hydrocephalus in patients with aneurysmal SAH but without hydrocephalus on admission.

The outcomes of hydrocephalus have been extensively studied. Hydrocephalus can result in long-term cognitive decline and the development of psycho-organic disorders [22,23]. This study demonstrates the worst short-term outcome and longest duration of hospitalization in patients with subsequent hydrocephalus, and the prognosis is also worst after 1.5 years of follow-up. Worse short- and long-term outcomes and longer duration of hospitalization are also noted in shunt-dependent hydrocephalus patients.

The current study has several limitations. First, it is a retrospective analysis and therefore subject to bias of unmeasured factors. Second, patients who were comatose or considered unlikely to survive for more than one week and had pre-existing neurologic deficits have been excluded. Third, hydrocephalus can occur in both the acute stage and later stages during treatment. The findings may underestimate the “true” frequency of hydrocephalus in asymptomatic patients. Thus, there is continued uncertainty in assessing the incidence of hydrocephalus after aneurysmal SAH in non-selected patients.

## Conclusions

The presence of intra-ventricular hemorrhage, lower mean score of Glasgow Coma Scale, and higher mean scores of the modified Fisher SAH and World Federation of Neurosurgical grading on admission imply risks of shunt-dependent hydrocephalus in patients without hydrocephalus on admission. These patients also have worse short- and long-term outcomes and longer hospitalization. More prospective multi-center investigations evaluating the role of hydrocephalus on outcome of aneurysmal SAH and timing of surgical intervention on this specific group of patients are warranted. Despite the high proportion of disability during the acute stage, adequate treatment of neurologic complications is essential for improving therapeutic outcomes.

## Competing interests

All authors declare that they have no competing interests.

## Authors’ contributions

All authors have read and approved the final manuscript. YMW and YJL had substantial contributions to conception and design, data acquisition and analysis, drafting the manuscript and revising the manuscript. THL, NTW, BCC, WCL, YJS, CCH, TMY, MJC, WNC, LHL had substantial contributions to conception and design, clinical data analysis. CHL and HCW had substantial contributions to conception and design, data analysis, critical revision and final approval of the revision.

## Pre-publication history

The pre-publication history for this paper can be accessed here:

http://www.biomedcentral.com/1471-2482/12/12/prepub
